# Knowledge and perception of tuberculosis and the risk to become treatment default among newly diagnosed pulmonary tuberculosis patients treated in primary health care, East Nusa Tenggara: a retrospective study

**DOI:** 10.1186/s13104-015-1209-6

**Published:** 2015-06-10

**Authors:** Ikhwanuliman Putera, Trevino A Pakasi, Elvina Karyadi

**Affiliations:** Faculty of Medicine, Universitas Indonesia, Jakarta, Indonesia; Department of Community Medicine, Faculty of Medicine, Universitas Indonesia - Cipto Mangunkusumo Hospital, Jl. Pegangsaan Timur No. 16, Cikini, 10320 Jakarta, Indonesia; Faculty of Medicine, South East Asia Minister of Education Organization (SEAMEO) TROPMED Regional Center of Community Nutrition, Universitas Indonesia, Jakarta, Indonesia; Micronutrient Initiative, 2nd Floor, Wirausaha Building, Jl. H.R. Rasuna Said Kav C-5, 12920 Jakarta, Indonesia

**Keywords:** Knowledge, Perception, Pulmonary tuberculosis, Primary health care, Treatment default

## Abstract

**Background:**

Despite the high efficacy of tuberculosis (TB) drug regiments, one of the barriers in the TB control program is the non-compliance to treatment. Morbidity, mortality, and risk to become resistant to drugs are emerging among defaulters. Thus, the aim of this study is to identify the factors, especially knowledge and perceptions of TB and association with treatment default among patients treated in primary care settings, East Nusa Tenggara.

**Methods:**

This study was part of a bigger cohort community-based controlled trial study. The subjects were newly diagnosed pulmonary TB patients from four districts in East Nusa Tenggara. Knowledge, perception of TB, and other related factors were assessed prior to the treatment. Patients who interrupted the treatment in two consecutive months were classified as defaulters, as World Health Organization stated. Odds ratio (OR) looking for factors associated with becoming defaulter was analyzed.

**Results:**

A total of 300 patients were recruited for this study. At the end of the treatment, 255 patients (85%) completed the treatment without interruption from regular visit. In univariate analysis, none of the socio-demographic factors attributed to treatment default yet lack of knowledge and incorrect perception of TB prior therapy (OR 2.49 1.30–4.79 95% CI, p = 0.006; OR 5.40 2.64–11.04 95% CI, p < 0.001, respectively). In multivariate analysis, only incorrect perception of TB showed significant association with treatment default (OR 4.75 2.30–9.86 95% CI).

**Conclusions:**

Assessing the knowledge and perception of TB prior to the treatment in newly pulmonary TB patients is important as both of them were known as risk factor for treatment default. Education and counseling may be required to improve patients’ compliance to treatment.

## Background

Tuberculosis (TB) continues as a major public health problem in Indonesia since the World health Organization (WHO) declared TB as a global health emergency. Despite the high efficacy of the drug regiments recommended by WHO, poor compliance to treatment has become one of the identified obstacles toward accomplishing the goal of TB control program. As a communicable disease, poor compliance to treatment could increase the risks of morbidity, mortality, and resistance to standard drugs [1].

An advance in strategies to improve the TB patients’ compliance refers to directly observed treatment short-course (DOTS) strategy [2, 3]. This strategy requires every TB patient to have a treatment-partner that should observe daily drug taking of the patients. Several factors have already been reported across different populations having association with noncompliance to treatment, including low income patients, alcoholism, HIV co-infection, male gender, homeless patients and low level of knowledge and interest toward treatment [4–6]. Because of inadequate duration of treatment, treatment default possess high possibility to acquire drug resistance [7]. However, as the drug susceptibility testing (DST) is not available universally, especially in primary care setting, patients are often retreated with first-line drugs [8]. In addition, study showed that retreatment of defaulters had lower success rate compared to treatment failure or relapse cases [9].

Indonesia is still among the high burden countries of TB, because its population is among the largest countries in the world [10]. It was estimated that the rate of new TB cases was approximately 244 per 100.000 people in 2010 [11]. The east region of Indonesia was the major contributor of TB patients, such as Papua and East Nusa Tenggara (NTT province) with high prevalence of TB compared to other provinces [12]. The majority of TB patients in NTT province use primary health care (Puskesmas) to access the TB diagnostic and treatment [11]. Unfortunately, in primary care, the available diagnostic tool is Ziehl–Neelsen staining which gives no information about drug susceptibility [13]. Meanwhile, drop out cases in Indonesia were reported as much as 47.9% of the total cases. Moreover, east region of Indonesia was the highest contributor to the drop out cases (59.7%) [14].

Previously, there was a controlled trial community based study done in the NTT province [15]. The study covered four different areas within the province, namely one municipality, two rural areas and one small island. Since the study deal with defaulters, we aimed to assess the association between knowledge, perception, and TB treatment default. Minimizing defaulters through identifying patients with risk factors will be valuable to decrease potency of TB drug resistance in primary care setting, especially in eastern region of Indonesia.

## Methods

### Study design and subjects

The subjects were part of a larger study entitled “Zinc and vitamin A supplementation fails to reduce sputum conversion time in severely malnourished pulmonary tuberculosis patients in Indonesia” [15]. It was a community-based controlled trial implemented in four districts in NTT province that worked with 22 primary health cares (Puskesmas). Its main outcome was sputum conversion time among pulmonary TB patients treated with additional micronutrient supplementation. Subjects were newly pulmonary tuberculosis patients aged 15–55 years old confirmed by positive sputum smear using the national guidelines for TB diagnosis and treatment [16]. Exclusion criteria were pregnant or lactating women and subjects with underlying chronic disease. Random allocation sampling was done with previously stated estimation sample size. A total of 300 patients were then treated using standard regiment of anti-tuberculosis given as 300 mg isoniazid, 450 mg rifampicin, 1,500 mg pyrazinamide and 750 mg ethambutol daily for 2 months, followed by 600 mg isoniazid and 450 mg rifampicin three times a week during the next 4 months. Beside anti-tuberculosis therapy, subjects were divided into four groups receiving additional supplementation of zinc alone, vitamin A alone, zinc + vitamin A, or placebo, prepared by Kimia Farma Ltd, Indonesia. Every patients had a treatment-partner to remember patients for daily drug taking and report any encountered problems to health care professionals during treatment. Adherence to treatment was assessed by presence to regular PHC visit. During the first 2 months of treatment, patients were asked to come every week to provide three-times sputum. Hereafter, patients then come on monthly regular basis. After completion of that study, we found some patients who become defaulter. Defaulter were defined as the patients interrupted for drug taking in two consecutive months [8]. As we had baseline data prior to the treatment, including socio-demographic characteristics, knowledge, and perception of TB, we then analyzed patients’ records retrospectively.

### Data collection

Prior to treatment, all patients recruited in previous RCT study [13] were interviewed for their socio-demographic profiles, knowledge and perception to TB, and symptoms of TB using semi-structured questionnaire. The interview was done using local language by nurses who were trained for the study’s purpose. The nurses were TB case managers in the PHC where the study took place.

### Knowledge and perception of TB

There were nine true–false statement to assess patients’ knowledge. These included several general theory regarding scientific basis of TB such as etiology, mode of transmission, diagnosis and treatment of TB. Perception was assessed using ten agree-disagree statements regarding patients’ subjective belief about their illness. Common misconception was due to local belief that perceived TB as incurable disease, caused by sin or curse, and ashamed what other might say for having this disease. Every correct answer on knowledge and correct perception about present illness scored 1 while the wrong one scored 0. Total score for knowledge and perception therefore range from 0 to 9 and 0 to 10, respectively. Higher score indicate better understanding and subjective belief. The score of knowledge and perception were then classified into either high (with minimum five statements answered correctly) or low (less than five statements answered correctly).

### Statistical analysis

Statistical analysis was done using SPSS® Statistics (Statistical Package for the Social Science, Inc., Chicago, USA) for Windows version 11.5. The association between the characteristics of subjects and treatment default was initially tested using Chi square test. All variables with initial of p < 0.2 were included in the multivariate regression model. Backward stepwise elimination procedures were performed to fit the final model with 95% confidence interval.

### Ethical approval

Informed consent was obtained from all subjects. The ethical approval for this study was obtained from the Ethical Committee of the Faculty of Medicine, Universitas Indonesia, Jakarta, Indonesia.

## Results

### Patients’ characteristics

Initially, 300 patients were recruited in the trial and 255 patients (85%) completed the treatment. The remaining patients were classified as defaulters as they interrupted the treatment. The median age for the entire subjects was 28 (15–59) years old and 188 of them (62.7%) were male. Overall, the number of subjects with low level of education was high: no schooling 51 (17%) and basic school 153 (51%). Table 1 shows the details about patients’ characteristics in both groups. There was no statistically significant association between any variable of patients’ characteristics and becoming treatment default.

**Table 1:** Characteristics of the TB patients

Variables	Treatment default (n = 45, %)	Finished treatment (n = 255, %)	Total	*P*
Sex				
Male	29 (64.4)	159 (62.4)	188 (62.7)	0.79
Female	16 (35.6)	96 (37.6)	112 (37.3)	
Age (median, IQR)	34 (24–45)	28 (22–39)	28 (15–59)	0.07
15–34 years old	24 (53.3)	171 (67.1)	195 (65.0)	0.19
35–54 years old	19 (42.2)	78 (30.6)	97 (32.3)	
>55 years old	2 (4.4)	6 (2.4)	8 (2.7)	
District				
Kupang city	19 (42.2)	96 (37.6)	115 (38.3)	0.68
Kupang district	14 (31.1)	76 (29.8)	90 (30.0)	
TTU district	7 (15.6)	60 (23.5)	67 (22.3)	
Rote-Ndao district	5 (11.1)	23 (9)	28 (9.3)	
Length of education				
No schooling	10 (22.2)	41 (16.1)	51 (17.0)	0.15
Finished 9 years	27 (60)	126 (49.4)	153 (51.0)	
Finished 12 years	4 (8.9)	55 (21.6)	59 (19.7)	
Higher education	4 (8.9)	33 (12.9)	37 (12.3)	
Marital status				
Single	16 (35.6)	118 (46.3)	134 (44.7)	0.35
Married	28 (62.2)	129 (50.6)	157 (52.3)	
Divorced/widowed	1 (2.2)	8 (3.1)	9 (3.0)	
History of smoking				
Yes	11 (24.4)	67 (26.3)	78 (26.0)	0.80
No	34 (75.6)	188 (73.7)	222 (74.0)	
Drinking alcohol				
Yes	12 (26.7)	97 (38.0)	109 (36.3)	0.14
No	33 (73.3)	158 (62.0)	191 (63.7)	
Indigenous				
Timor/Rote	32 (71.1)	193 (75.7)	225 (75.0)	0.51
Non-indigenous	13 (28.9)	62 (24.3)	75 (25.0)	
AFB level				
+	13 (28.9)	76 (29.8)	89 (29.7)	0.99
++	14 (31.1)	77 (30.2)	91 (30.3)	
+++	18 (40)	102 (40)	120 (40.0)	

*  Variables with p < 0.2 were included in multivariate logistic regression analysis.

### Knowledge and perception of tuberculosis

The summary of knowledge and perception of TB in both groups is shown in Tables 2 and 3, respectively. The median score of knowledge among finished treatment group was 8 (6–9) compared to 6 (2.25–8) among defaulters. For perception TB, the median score among finished treatment group was 9 (7–10) compared to 8 (2.5–10) among defaulters.

**Table 2:** Knowledge of tuberculosis

Variables	Treatment default (n, %)	Finished treatment (n, %)	Odds ratio to become dropout (95% CI)	*p*
Score knowledge*	6 (2.25–8)	8 (6–9)		
The cause of TB is bacteria				0.007
Correct	5 (11.1)	82 (32.2)	3.8 (1.4–9.9)	
Incorrect	40 (88.9)	173 (67.8)		
TB is a curable disease				
Correct	34 (75.6)	232 (91.0)	3.3 (1.5-7.3)	0.004
Incorrect	11 (24.4)	23 (9.0)		
TB is a contagious disease				
Correct	31 (68.9)	226 (88.6)	3.5 (1.7–7.4)	0.001
Incorrect	14 (31.1)	29 (11.4)		
TB is an airborne disease				
Correct	16 (35.6)	154 (60.4)	2.8 (1.4–5.3)	0.003
Incorrect	29 (64.4)	101 (39.6)		
TB patient should dispose sputum in a close container				
Correct	24 (53.3)	180 (70.6)	2.1 (1.1–4.0)	0.024
Incorrect	21 (46.7)	75 (29.4)		
Closing mouth while coughing prevents transmission to others				
Correct	16 (35.6)	148 (58.0)	2.5 (1.3–4.8)	0.006
Incorrect	29 (64.4)	107 (42.0)		
TB is easily spread in a crowded house				
Correct	25 (55.6)	194 (76.1)	2.5 (1.3–4.9)	0.005
Incorrect	20 (44.4)	61 (23.9)		
TB should be treated for at least 6 months				
Correct	28 (62.2)	201 (78.8)	2.2 (1.2–4.4)	0.018
Incorrect	17 (37.8)	54 (21.2)		
TB is diagnosed by sputum examination				
Correct	29 (64.4)	225 (88.2)	4.1 (2.0–8.5)	<0.001
Incorrect	16 (35.6)	30 (11.8)		

* Data are presented as median and IQR, Chi Square test, significant at p < 0.05.

**Table 3:** Perceptions of tuberculosis

Variables	Treatment default (n, %)	Finished treatment (n, %)	Odds ratio to become dropout (95% CI)	*P*
Score perception of TB*	8 (2.5–10)	9 (7–10)		
TB is a life threatening disease for me				
Agree	31 (68.9)	227 (89.0)	3.7 (1.7–7.7)	<0.001
Disagree	14 (31.1)	28 (11.0)		
TB is caused by a curse				
Agree	21 (46.7)	60 (23.5)	2.8 (1.5–5.5)	0.002
Disagree	24 (53.3)	195 (76.5)		
I have made a sin therefore I got TB				
Agree	22 (48.9)	61 (23.9)	3.0 (1.6–5.8)	<0.001
Disagree	23 (51.1)	194 (76.1)		
I am ashamed because I got TB				
Agree	26 (57.8)	97 (38.0)	2.2 (1.2–4.2)	0.015
Disagree	19 (42.2)	158 (62.0)		
TB treatment is available at Puskesmas				
Agree	29 (64.4)	224 (87.8)	4.0 (198–8.2)	<0.001
Disagree	16 (35.6)	31 (12.2)		
TB treatment is expensive for me				
Agree	21 (46.7)	70 (27.5)	2.3 (1.2–4.4)	0.011
Disagree	24 (53.3)	185 (72.5)		
TB patients must be isolated from the community				
Agree	20 (44.4)	60 (23.5)	2.6 (1.4–5.0)	0.004
Disagree	25 (55.6)	195 (76.5)		
TB needs a serious treatment				
Agree	31 (68.9)	231 (90.6)	4.3 (2.0–9.3)	<0.001
Disagree	14 (31.1)	24 (9.4)		
I have to follow the treatment routinely				
Agree	31 (68.9)	239 (93.7)	6.7 (3.0–15.1)	<0.001
Disagree	14 (31.1)	16 (6.3)		
I can be cured if treated in Puskesmas				
Agree	31 (68.9)	234 (91.8)	5.0 (2.3–10.9)	<0.001
Disagree	14 (31.1)	21 (8.2)		

* Data are presented as median and IQR, Chi Square test, significant at p < 0.05.

There was a significant difference of knowledge score between both finished treatment and treatment default groups (OR 2.49; 1.30–4.79 95% CI, p = 0.006). Moreover, when answers from each question were analyzed, there were statistically significant difference of proportion of correct answers between both groups (p < 0.05). Similarly, when each item of perception of TB was assessed, all answers indicative for misconception were found to be significantly associated with treatment default (p < 0.05). The odds ratio having low perception category to become defaulter was 5.40 (OR 2.64–11.04 95% CI, p = <0.000, Table 4).

**Table 4:** Odds ratio (OR) of knowledge and perception category to become treatment default

Variables	Treatment default (n, %)	Finished treatment (n, %)	OR (95% CI)	*P*
Category of TB knowledge score				
Low (0–5)	20 (44.4)	62 (24.3)	2.49 (1.30–4.79)	0.006
High (≥6)	25 (55.6)	193 (75.7)		
Category of perception of TB score				
Low (0–5)	18 (40.0)	28 (11.0)	5.40 (2.64–11.04)	<0.001
High (≥6)	27 (60.0)	227 (89.0)		

In the multivariate analysis, we selected the length of education, alcohol status, the category of age, the category of knowledge, and the category of perception of TB to be entered in logistic regression model. Low category of perception of TB was the only important risk factor toward treatment default with adjusted OR of 4.75 (2.30–9.86, 95% CI; p < 0.001, Table 5).

**Table 5:** Multivariate logistic regression model to become treatment default

Variables	Adjusted OR (95% CI)	*P*
Low education (no schooling or only finishing elementary school)	2.07 (0.90–4.74)	0.09
Low category of perception of TB	4.75 (2.30–9.86)	<0.001

## Discussion

This study showed that none of the patients’ characteristics and socio-demographic factors had an association with completion of TB treatment. Instead, knowledge and perception of TB played a major role in the treatment compliance. The treatment default was relatively acceptable in our subjects (15%) since the indicator of success rate of treatment was 85% [11]. All finished treatment had been previously described to have negative sputum smear after completion of treatment [15]. Compared with education that previously reported by Indonesian Ministry of Health to have a contribution to patient’s compliance [11], our study emphasized the importance of both knowledge and perception of TB.

Only 5 patients (11.4%) in defaulters answered correctly that TB is caused by bacteria, compared to 82 patients (32.2%) to the finished treatment group. The defaulters also showed poor knowledge about how TB spreads in community, whether TB is curable or not, and how long the treatment is. Regarding the perception of TB, only 31 patients (68.9) perceived that they can be cured if they treated in PHC and compliance to treatment is important in TB treatment. As mentioned before, primary health care (Puskesmas) is the frontier of health service in Indonesia so that the knowledge and perception about the usefulness of health services are beneficial for patients.

Overall, the perception of TB in our subjects was fairly good. Many of the subjects no longer believed that TB is caused by curse, sins, or feel ashamed with their neighborhood (Table 2). The perception of TB was important especially concerning compliance, the role of PHC, and treatment motivation. Our findings also support previous studies aimed to describe the knowledge and perception in TB patients. Perception and knowledge are two important factors toward the completion of TB therapy and thus health personnel in primary health care have to be aware about these two issues [17–19].

Patient’s related factors play an important part in patient’s compliance. Ignorance to treatment adherence and low knowledge about TB were reported in another study to become the contributors for treatment default. Several reasons why such patients had poor knowledge were no history of TB in family and never reading or attending public education about TB [4]. In other studies, several factors were reported having significant association with non-compliant patients, such as alcoholism [4, 5], relatively high cost and time to come to the health facilities [19], having a family member with TB [19], age and marital status [4], and low monthly income [4].

Our study encountered some limitations. We could not obtain the data about the exact reason why patients become treatment default as more precise information will necessary for the next intervention. Patients might die or be transferred to other TB program facilities. However, this study was conducted in the primary health care where patients encountered medical problem daily. Primary heath care facilities become the frontier of medical service in Indonesia so that it might face the real public health problems. Further, we also could not record the adherence as we had no data about the diary of drug taking at home.

## Conclusions

In East Nusa Tenggara setting, DOTS treatment strategy was already implemented for years. Since this area contributes to the high number of TB cases, completion to treatment has to be enhanced to reduce the morbidity, mortality, disease spread in community, and resistance to drug regiments. Primary health center (Puskesmas) must be able to recognize pulmonary TB patients with high probability of becoming treatment default. In this study, assessing the knowledge, and more important the perception of TB, only required a simple method but it was useful to recognize such patients with higher risk. Giving education and counseling may enhance the compliance of the patients.
